# Circulating miRNAs as molecular markers of occupational grain dust exposure

**DOI:** 10.1038/s41598-020-68296-5

**Published:** 2020-07-09

**Authors:** Anne Straumfors, Nur Duale, Oda A. H. Foss, Steen Mollerup

**Affiliations:** 1 National Institute of Occupational Health, Gydas vei 8, PO Box 5330, 0304 Majorstuen, Oslo, Norway; 2 Department of Molecular Biology, Norwegian Institute of Public Health, PO Box 222, 0213 Skøyen, Oslo, Norway

**Keywords:** miRNAs, Molecular biology, Biomarkers

## Abstract

Dust from grain and feed production may cause adverse health effects in exposed workers. In this study we explored circulating miRNAs as potential biomarkers of occupational grain dust exposure. Twenty-two serum miRNAs were analyzed in 44 grain dust exposed workers and 22 controls. Exposed workers had significantly upregulated miR-18a-5p, miR-124-3p and miR-574-3p, and downregulated miR-19b-3p and miR-146a-5p, compared to controls. Putative target genes for the differentially expressed miRNAs were involved in a range of Kyoto Encyclopedia of Genes and Genomes signaling pathways, and ‘Pathways in cancer’ and ‘Wnt signaling pathway’ were common for all the five miRNAs. MiRNA-diseases association analysis showed a link between the five identified miRNAs and several lung diseases terms. A positive correlation between miR-124-3p, miR-18a-5p, and miR-574-3p and IL-6 protein level was shown, while miR-19b-3p was inversely correlated with CC-16 and sCD40L protein levels. Receiver-operating characteristic analysis of the five miRNA showed that three miRNAs (miR-574-3p, miR-124-3p and miR-18a-5p) could distinguish the grain dust exposed group from the control group, with miR-574-3p as the strongest predictor of grain dust exposure. In conclusion, this study identified five signature miRNAs as potential novel biomarkers of grain dust exposure that may have potential as early disease markers.

## Introduction

Workers at grain elevators and animal feed mills are exposed to grain dust containing a complex mixture of inorganic and organic particles from soil, plants, insects and microorganisms, and includes components such as endotoxins, β-1,3-glucans and mycotoxins^1,2^. The individual dust components have the ability to induce inflammatory and allergic responses, and symptoms from the airways are frequently observed among grain handlers^3–8^. Endotoxin has been proposed as a target for measurements of occupational health hazard of grain dust^9^, but depending on the grain dust composition, other components in the dust may be stronger associated with health effects. Diverging exposure–response relationships have indeed been observed between studies^2,3,10–12^.

Although exact mechanisms are not completely elucidated, the pathological response to grain dust exposure may include activation of inflammatory and allergic pathways. miRNAs are small non-coding RNA molecules that regulate gene expression by binding to specific mRNAs, which leads to either degradation of the mRNA or inhibition of translation, thus suppressing gene activity^13^. Several studies have suggested that the toxicity of environmental chemicals are mediated by miRNAs^14^, and particular miRNA profiles have been associated with different diseases, such as asthma, COPD and cancer^15^. Little is known about the miRNA induction following grain dust exposure. However, it is shown that miR-146a are induced by LPS and other proinflammatory mediators such as TNFα, IL-1β and CD40L^16^. Epigenetic studies in mice models suggests a role for increased miR-146a in allergic rhinitis^17^. Reduced expression of miR-146a and let-7c have been associated with inflammation and the progressive phase of COPD^18,19^, whereas increased expression of miR-126 and miR-26c compared with controls are reported in COPD phase III^20^. Several asthma models have shown increased expression of miR-21, and furthermore, that this is connected with the expression of IL-12 and a Th2 response, including models with *A. fumigatus*^15^. Some miRNAs have therefore gained status as promising biomarkers for different diseases. MiRNAs circulates in a stable, cell-free form in the bloodstream, and the abundance of specific miRNAs in plasma or serum can serve as biomarkers. Epigenetic changes closely linked to grain dust exposure or mechanistically linked to health outcomes, may shed light on the biological mechanisms of exposure–response, and result in biomarkers of exposure or effect.

The primary objectives of this study was to investigate whether workers exposed to grain dust and its components have different expression profiles of circulating serum miRNA compared with controls, and to explore the potential of differentially expressed miRNAs as possible biomarkers of exposure and health effects. Secondary objectives were to investigate how the miRNAs were associated to (1) the serum levels of proteins involved in inflammation and platelet activation, and to (2) exposure to dust from grain elevators and compound feed mills, including bacteria, endotoxin, fungal spore and 1,3-β-glucans (bioaerosol exposure).

## Results

The lung function and distribution of age and body size parameters in the exposed workers and controls was similar, whereas the prevalence of current cold were higher in the controls, and the prevalence of farm childhood and currently living at a farm were higher among controls (Table 1). Only 21% of the exposed workers reported to use respiratory protective equipment (RPE).

**Table 1:** Characteristics of the study population of grain and animal feed industry workers in Norway, including 44 grain dust exposed workers and 22 assumed unexposed administrative workers as controls.

	Exposed workers (*n* = 44)	Controls (*n* = 22)
Age (year)^a^	45 (16–61)	49 (31–60)
Height (cm)^a^	180 (162–195)	178 (160–191)
Weight (kg)^a^	89 (63–121)	90 (58–99)
Body mass index (kg/m^2^)^a^	27 (22–36)	27 (23–32)
Cold (in %)	19	27
Farm childhood (in %)	36	46
Living at a farm (in %)	23	32
RPE use (in %)	21	0
FVC % of predicted at baseline^a^	94 (66–133)	99 (82–122)
FEV_1_% of predicted at baseline^a^	91 (68–127)	94 (71–120)

*RPE* respiratory protective equipment, *FVC* forced vital capacity, *FEV*_*1*_ forced expired volume in the first second.

^a^Median (min–max).

### Bioaerosol exposure

The high endotoxin exposure of GM 439 EU/m^3^ exceeded the health-based recommended occupational exposure limit (OEL) of 90 EU/m^3^ by more than fourfold (Table 2). The mean exposures of inhalable dust, fungal spores, bacteria and β-1,3-glucans were moderate, but large variances were observed between workers, and existing and recommended OELs of other bioaerosol components were exceeded in 7–18% of the samples depending on the exposure component (Table 2).

**Table 2:** Full-shift bioaerosol exposure of grain and animal feed industry workers in Norway.

Exposure parameter	*n*	AM	GM	GSD	Above OEL (%)
Grain dust (mg/m^3^)	44	1.6	0.9	3.00	7
Endotoxin (EU/m^3^)	43	1,252	439	5.15	84
β-1 → 3-glucan (µg/m^3^)	44	14.7	6.4	4.49	No OEL
Bacteria (counts/m^3^)	44	52 × 10^4^	20 × 10^4^	5.53	14
Fungal spores counts/m^3^)	44	7.8 × 10^4^	3.5 × 10^4^	3.30	18

*AM* arithmetic mean, *GM* geometric mean of log10 transformed values, *GSD* geometric standard deviation, *OEL* occupational exposure limit. Norwegian OELs: grain dust (organic dust) = 5 mg/m^3^; endotoxins = 90 EU/m^3^ (health-based recommended); bacteria = 10^6^ counts/m^3^ (suggested based on lowest observed effect levels); fungal spores = 10^5^ counts/m^3^ (suggested based on lowest observed effect levels).

### Serum proteins

The concentration of pneumoproteins and markers of inflammation and platelet activation is shown in Table 3. The concentration of CC-16 and IL-6 was significantly higher in exposed workers than the controls, whereas the concentration of the other proteins was similar in the two groups.

**Table 3:** Serum protein concentrations of dust exposed workers and unexposed controls in the Norwegian grain and animal feed industry.

	Exposed workers (*n* = 44)		Controls (*n* = 22)		Group differences	
	GM (GSD)	GM_adj_ (GSE)	GM (GSD)	GM_adj_ (GSE)	*p *value	*p *value_adj_
***CC-16 (ng/mL)***	**5.1 (1.4)**	–	**3.9 (1.4)**	–	**0.004**	**–**
SP-D (ng/mL)	99.7 (1.7)	105 (1.1)	101 (1.9)	112 (1.1)	0.9	0.6
SP-A (µg/mL)	24.3 (11.7)	-	38 (5.8)	–	0.4	–
***IL-6 (pg/mL)***	**1.8 (1.8)**	**1.8 (1.1)**	**1.2 (1.7)**	**1.2 (1.1)**	**0.01**	**0.004**
TNF-α (pg/mL)	1.1 (1.3)	1.1 (1.1)	1.1 (1.4)	1.1 (1.1)	0.9	0.9
Fibrinogen (mg/mL)	3.3 (1.4)	–	3.8 (1.3)	–	0.1	**–**
CRP (mg/L)	1.5 (2.9)	1.4 (1.2)	1.3 (2.7)	1.3 (1.2)	0.7	0.8
sCD40L (ng/mL)	2.0 (2.5)	1.9 (1.2)	1.5 (2.7)	1.6 (1.2)	0.3	0.4
sP-selectin (ng/mL)	52.7 (1.6)	–	51 (1.7)	–	0.8	–

*GM* geometric mean, *GSD* geometric standard deviation, *GM*_*adj*_ geometric mean adjusted for confounders, *GSE* standard error of GM_adj_; adjustments. *SP-D* % body fat and farm childhood, *IL-6*% body fat, *TNF-α* age and % body fat, *CRP* age and % body fat, *sCD40L* % body fat.

Differences in the GM between exposed workers and controls were tested by independent sample t tests. Significant different concentrations, as judged by a *p* value ≤ 0.05, are highlighted in bold.

### Serum miRNA expression profiles in grain and animal feed workers

**Figure 1:**
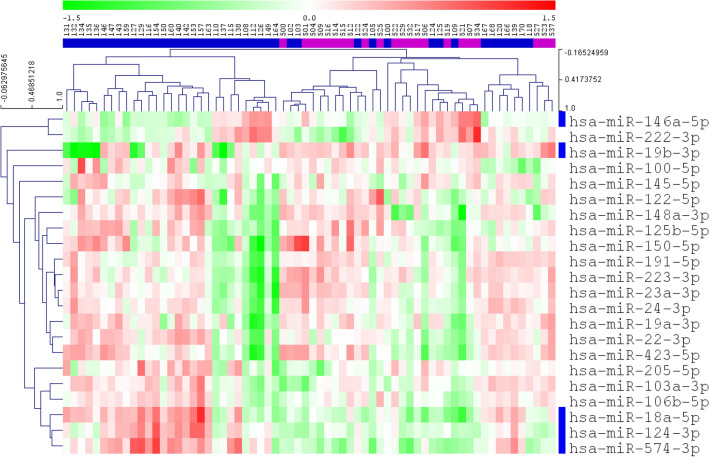
Heat map of miRNA expression profile in exposed workers and controls. Unsupervised hierarchical clustering analysis of the relative expression level of 22 miRNAs (measured by qPCR), and the clustering analysis is based on similarities in miRNA expression pattern. Red pixels represent up-regulated miRNAs whereas green pixels represent down-regulated miRNAs. Samples are horizontally labelled based on the exposure group they belong to (blue: exposed group and purple: control group). Vertically, blue labelled miRNAs (*n* = 5) indicate significantly differentially expressed miRNAs. Data are presented as log2-transformed normalized relative expression values.

From the results of initial miRNA screening, 22 miRNAs with over ± twofold mean expression level difference between pools were identified, and subjected to individual validation of expression levels. Hierarchical clustering analysis (average linkage and Euclidean distance similarity measurement) of these 22 miRNAs showed that samples from most of the exposed workers clustered close to each other, while samples from the control workers and some samples from the exposed workers clustered together (Fig. 1).

**Figure 2:**
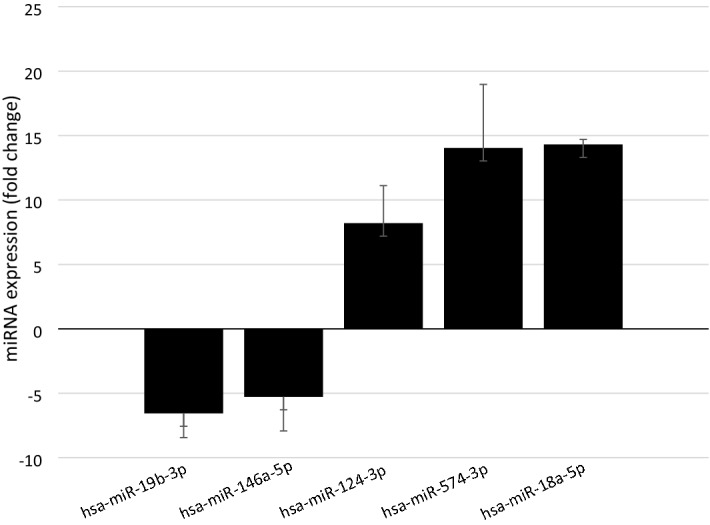
Fold change (SE) of miRNAs in exposed workers relative to controls. Only miRNA significantly different between exposed and controls are shown.

The individual sample analysis identified five miRNAs that were significantly differentially expressed between exposed workers and controls (hsa-miR-124-3p, hsa-miR-146a-5p, hsa-miR-18a-5p, hsa-miR-19b-3p and hsa-miR-574-3p) (Fig. 2). The miRNA expression level in exposed workers differed from controls as shown by fold change in Fig. 2. The expression level of hsa-miR-124-3p, hsa-miR-18a-5p, and hsa-miR-574-3p was significantly upregulated in exposed workers compared with controls (*p* ≤ 0.001 for all), also shown by the clustering of theses miRNAs close to each other in a sub-cluster (Fig. 1), whereas hsa-miR-19b-3p and hsa-miR-146a-5p were downregulated in the exposed group compared with the controls (*p* = 0.001 and *p* = 0.045, respectively, Fig. 2).

### In silico analysis of significantly altered miRNAs

To identify biochemical signaling pathways that had been affected by grain dust exposure induced miRNA expression changes, we analyzed predicted target genes of the five significantly affected miRNAs. The KEGG enrichment analysis indicated that the five significantly differentially expressed miRNAs (hsa-miR-124-3p, hsa-miR-146a-5p, hsa-miR-18a-5p, hsa-miR-19b-3p and hsa-miR-574-3p) targeted genes were involved in a range of signaling pathways (Table 4). A total of 20 KEGG signaling pathways were significantly enriched. Two KEGG pathways (‘Pathways in cancer’ and ‘Wnt signaling pathway’) were common for all the five miRNAs. Target genes for the two upregulated miRNAs, hsa-miR-124-3p and hsa-miR-574-3p were enriched in thirteen and twelve pathways, respectively (Table 4). Among the downregulated miRNAs, target genes for hsa-miR-146a-3p were enriched in eleven pathways (Table 4).

**Table 4:** List of KEGG signaling pathways involving the five differentially expressed miRNAs predicted by their putative target genes.

Pathway name	# predicted target genes	# genes	*p *value	FDR
**Upregulated miRNAs**				
hsa-miR-574-3p (n = 12)				
Axon guidance	97	129	1.85E−08	3.59E−06
Neurotrophin signaling pathway	93	129	1.00E−06	1.90E−04
Endocytosis	128	187	1.18E−06	2.24E−04
MAPK signaling pathway	178	272	1.27E−06	2.41E−04
* Pathways in cancer*	207	330	1.41E−05	2.60E−03
Long term potentiation	54	71	1.49E−05	2.75E−03
ErbB signaling pathway	65	89	2.11E−05	3.85E−03
* Wnt signaling pathway*	102	152	4.98E−05	8.96E−03
T cell receptor signaling pathway^a^	76	110	1.03E−04	1.84E−02
Apoptosis	62	87	1.09E−04	1.92E−02
Chemokine signaling pathway	122	189	1.37E−04	2.43E−02
Non small cell lung cancer	41	54	1.71E−04	3.01E−02
hsa-miR-124-3p (n = 13)				
* Pathways in cancer*	295	330	7.16E−08	1.39E−05
Adherens junction	73	76	8.44E−08	1.63E−05
Axon guidance	121	129	1.20E−06	2.32E−04
Endocytosis	170	187	3.32E−06	6.34E−04
Fc gamma R mediated phagocytosis^a^	92	97	5.87E−06	1.12E−03
Small cell lung cancer	80	84	1.52E−05	2.84E−03
Gap junction	85	90	2.29E−05	4.25E−03
Ubiquitin mediated proteolysis	122	134	6.88E−05	1.21E−02
Regulation of actin cytoskeleton^a^	187	212	1.22E−04	2.10E−02
Neurotrophin signaling pathway	117	129	1.49E−04	2.57E−02
Long term potentiation	67	71	1.94E−04	3.32E−02
* Wnt signaling pathway*	136	152	2.24E−04	3.81E−02
Chemokine signaling pathway	167	189	2.25E−04	3.83E−02
Insulin signaling pathway	125	139	2.34E−04	3.98E−02
hsa-miR-18a-5p (n = 4)				
* Pathways in cancer*	313	330	1.81E−05	3.51E−03
Chemokine signaling pathway	182	189	6.00E−05	1.16E−02
* Wnt signaling pathway*	147	152	1.46E−04	2.78E−02
Ubiquitin mediated proteolysis	130	134	2.10E−04	3.99E−02
**Downregulated miRNAs**				
hsa-miR-146a-5p (n = 11)				
* Pathways in cancer*	293	330	1.81E−13	3.51E−11
Adherens junction	73	76	8.44E−08	1.63E−05
* Wnt signaling pathway*	135	152	6.04E−07	1.16E−04
Small cell lung cancer	78	84	2.28E−06	4.31E−04
MAPK signaling pathway	228	272	4.98E−06	9.37E−04
Apoptosis	79	87	1.97E−05	3.64E−03
ErbB signaling pathway	80	89	4.46E−05	8.03E−03
Gap junction	80	90	1.16E−04	2.03E−02
Ubiquitin mediated proteolysis	115	134	1.49E−04	2.59E−02
Non-small cell lung cancer	50	54	2.07E−04	3.57E−02
TGF beta signaling pathway^a^	76	86	2.70E−04	4.62E−02
hsa-miR-19b-3p (n = 7)				
* Pathways in cancer*	287	330	4.34E−09	8.43E−07
* Wnt signaling pathway*	134	152	1.24E−05	2.36E−03
Endocytosis	162	187	1.70E−05	3.23E−03
Ubiquitin mediated proteolysis	118	134	4.60E−05	8.65E−03
Insulin signaling pathway	121	139	1.22E−04	2.23E−02
Small cell lung cancer	76	84	1.35E−04	2.45E−02
Adherens junction	69	76	2.12E−04	3.79E−02

All five significantly differentially expressed miRNA target genes are enriched in pathways highlighted in italics.

^a^Pathway enriched only in one miRNA; KEGG (Kyoto Encyclopedia of Genes and Genomes). The KEGG enrichment analyses is based on hypergeometric statistical tests, including Benjamini and Hochberg (FDR < 0.05) multiple test adjustment.

miRNA-disease association analyses revealed that the lung disease terms ‘lung cancer’, ‘lung adenocarcinoma’, ‘lung cancer’, ‘lung disease’, ‘lung small cell carcinoma’, were associated with all the five differently expressed miRNAs, while terms like ‘asthma’, ‘chronic obstructive pulmonary disease’ or ‘pulmonary fibrosis’, were associated with some of the five identified miRNAs (Table 5).

**Table 5:** Associations between diseases and the five differentially expressed miRNAs extracted from the MNDR database.

Disease name	miRNA									
	hsa-miR-146a-5p		hsa-miR-19b-3p		hsa-miR-574-3p		hsa-miR-124-3p		hsa-miR-18a-5p	
	CS	Class	CS	Class	CS	Class	CS	Class	CS	Class
Lung cancer	0.4752	W	0.8939	S	0.8939	S	0.8939	S	0.8939	S
Non-small cell lung carcinoma	0.4752	W	0.7606	S	0.7311	S	0.8808	S	0.7606	S
COPD	0.8939	S	0.1097	P					0.1097	P
Asthma	0.8589	S	0.1097	P			0.1097	P	0.1097	P
Inflammation	0.7311	S	0.1097	P			0.1097	P	0.1097	P
Lacunar stroke	0.7311	S	0.4752	W	0.4752	W	0.7311	S		
Carotid stenosis									0.8808	S
Coronary artery disease	0.1097	P	0.9443	S			0.1097	P	0.7606	S
Atherosclerosis	0.9526	S	0.5725	W			0.1097	P	0.1097	P
Pulmonary hypertension	0.9751	S								
Intermediate coronary syndrome	0.1097	P	0.8939	S			0.1097	P	0.1097	P
Sepsis	0.7311	S			0.4752	W			0.1097	P
Adenoviridae infections									0.7311	S
Nasopharynx carcinoma							0.7606	S		

Confidence score (CS) for miRNA-disease association range from 0.110 to 0.975. Confidence score ranges between 0 and 1; and only well-supported miRNA–disease associations obtain a value close to 1. Class denotes the evidence type for each miRNA-disease association with S representing strong experimental evidence, W representing weak experimental evidence, and P representing prediction evidence; MNDR (Mammalian NcRNA-Disease Repository).

### Associations between serum miRNAs and exposure measurements

There was a statistical significant upregulation of hsa-miR-124-3p, hsa-miR-18a-5p and hsa-miR-574-3p (MANOVA F (3,62) = 9.6 *p* < 0.001, Wilkes’ ƛ = 0.62, partial ƞ^2^ = 0.32) and a statistical significant downregulation of hsa-miR-19b-3p and hsa-miR-146a-5p (MANOVA F (2,63) = 9.2 *p* < 0.001, Wilkes’ ƛ = 0.77, partial ƞ^2^ = 0.23) in exposed workers compared with unexposed workers. The miRNAs that differed significantly between exposed workers and controls were not associated with any of the bioaerosol exposure components, neither as continuous exposure variables nor as categorical (high and low concentration) exposure variables (results not shown). An effect of not wearing RPE was observed in general linear modeling of the upregulated miRNAs, where the upregulated conditions in the exposed group were strengthened by including the RPE-variable (from 2 to 6% increase in the explained variance).

### Associations between serum miRNAs and serum proteins

We evaluated the association between the five significantly differentially expressed miRNA and the nine serum proteins by determining the correlations between them. There were significant positive correlation between hsa-miR-124-3p, hsa-miR-18a-5p, and hsa-miR-574-3p expression levels and serum IL-6 level (Table 6). Further, there were significant inverse correlation between the hsa-miR-19b-3p expression level and serum CC-16 or sCD40L levels*.* No significant correlation was observed between hsa-miR-146a-5p and the nine serum proteins.

**Table 6:** Pearson correlations between serum miRNA and serum proteins. Log-transformed unadjusted values (*n* = 66).

	hsa-miR-146a-5p		hsa-miR-124-3p		hsa-miR-18a-5p		hsa-miR 19-3p		hsa-miR-574-3p	
	r_p_	*p*	r_p_	*p*	r_p_	*p*	r_p_	*p*	r_p_	*p*
***CC-16***	0.16	0.19	− 0.04	0.76	− 0.05	0.68	− **0.41**	**0.001**	0.05	0.71
SP-D	0.06	0.62	0.14	0.28	0.16	0.21	− 0.28	0.82	0.16	0.21
SP-A	0.06	0.62	− 0.11	0.39	− 0.15	0.23	− 0.19	0.12	− 0.06	0.66
***IL-6***	− 0.22	0.07	**0.38**	**0.001**	**0.33**	**0.008**	− 0.12	0.36	**0.34**	**0.005**
TNF-α	− 0.08	0.52	0.19	0.13	0.08	0.51	0.1	0.42	0.19	0.13
Fibrinogen	0.01	0.93	− 0.008	0.95	0.004	0.98	0.18	0.14	− 0.08	0.53
CRP	− 0.06	0.63	0.1	0.41	0.09	0.47	− 0.12	0.35	0.16	0.21
sp-selectin	− 0.07	0.6	− 0.11	0.38	− 0.06	0.62	− 0.0	0.73	0.07	0.56
***sCD40L***	0.13	0.28	− 0.17	0.18	− 0.15	0.23	− **0.24**	**0.05**	− 0.14	0.26

Bold values represent correlations significant at a *p* value ≤ 0.05.

We then searched whether the genes of the three significantly correlated proteins (IL-6, CC-16 and sCD40L) are target genes of the five differentially expressed miRNAs (hsa-miR-124-3p, hsa-miR-18a-5p, hsa-miR-574-3p, hsa-miR-19b-3p, hsa-miR-146a-5p). We found that the IL-6 and sCD40L genes were targeted by all five miRNAs, while the CC-16 gene was targeted by tree miRNAs (hsa-miR-124-3p, hsa-miR-18a-5p and hsa-miR-19b-3p).

### ROC analysis

**Figure 3:**
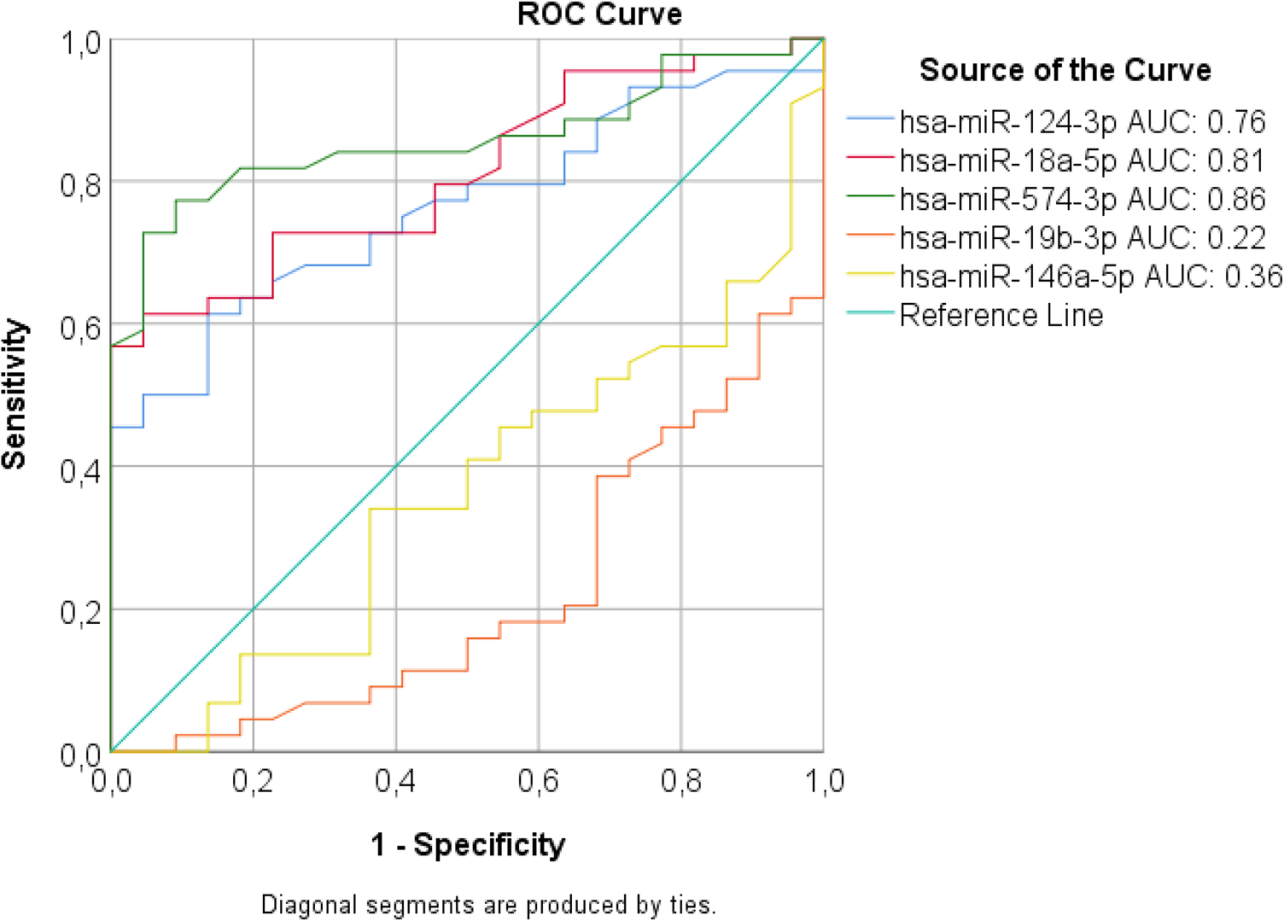
Receiver operating characteristic (ROC) curves with corresponding area under curve (AUC) statistics for circulating serum miRNAs to discriminate between grain dust exposed workers and controls.

We constructed receiver-operating characteristic (ROC) curves for each of the five significantly differentially expressed miRNAs using the relative expression values in order to assess the discriminatory values of these miRNAs; i.e. if any of these miRNAs have potential to discriminate between exposed and controls. The ROC analysis revealed that three miRNAs out of the five identified miRNAs could discriminate grain dust exposed workers from unexposed controls (Fig. 3). The area under the curve (AUC) of hsa-miR-574-3p was 0.858 (95% CI 0.769–0.948, *p* = 0.001) with 81.8% sensitivity and 77.3% specificity (Fig. 3). The AUC of hsa-miR-18a-3p was 0.810 (95% CI 0.710–0.911, *p* = 0.001) with 72.7% sensitivity and 77.3% specificity. The AUC of hsa-miR-124-3p was 0.763 (95% CI 0.651–0.876, *p* = 0.01) with 68.2% sensitivity and 63.6% specificity (Fig. 3). The ROC analysis indicated that hsa-miR-574-3p was the best predictor and may act as a potential molecular marker of grain dust exposure.

## Discussion

In this study, we performed circulating serum miRNA analysis of workers exposed to grain dust. In the initial screening, the expression profile of 84 miRNAs known to be found in serum were analyzed in pooled samples from 44 grain dust exposed workers and 22 unexposed controls in the grain industry. From this screening analysis, 22 differentially expressed miRNAs were identified. The 22 miRNA were validated in all individual samples (i.e., 44 grain dust exposed workers and 22 unexposed controls). Of the 22 individually tested miRNAs, five miRNAs were identified as differentially expressed between grain dust exposed workers and unexposed controls. Potential genes targeted by the significantly affected miRNAs were involved in totally 20 significantly enriched KEGG signaling pathways, and included genes for IL-6 and the pneumoprotein CC-16. Interestingly, the IL-6 and CC-16 proteins were present at significantly higher concentrations in the serum of exposed workers compared with controls. As the miRNAs also were potentially associated with several lung diseases, this study suggests that in addition to potential markers of grain dust exposure, some of these miRNA may have a potential as early disease markers in grain dust exposed workers.

In silico analysis of the five identified miRNAs showed that the target genes of these miRNAs were involved in signaling pathways that are implicated in the development of various types of lung diseases. The most enriched KEGG pathways such as pathways in cancer, MAPK kinase, apoptosis, chemokine, non-small cell lung cancer, adherence junction, small cell lung cancer, gap junction, ErbB, and TGFβ signaling pathways are related to cancer. Cancer may not be an end point per se, but may share common alterations and mechanisms with diseases such as COPD, asthma, and pulmonary fibrosis^21–23^. Enriched KEGG pathways like Wnt signaling, MAPK signaling, T cell receptor signaling, endocytosis and chemokine signaling, which all are important pathways in inflammatory and immunological responses, are particularly relevant for responses to occupational grain dust exposure, and may provide a potential mechanistic link between grain dust exposure and subsequent adverse health outcomes. The target genes for the five significantly differentially expressed miRNAs were all enriched in the Wnt signalling pathway. Wnt signals are active in early development and during the growth and maintenance of various tissues. Wnt proteins regulate the proliferation of cells, acting via receptor mediated intracellular signal. Abnormal Wnt signaling is involved in various human diseases, ranging from inflammation and degenerative diseases to cancer^24^. Thus, altered Wnt signaling might be implicated in the respiratory symptoms and inflammatory responses observed in grain workers^3,25,26^.

MiRNA-disease association analysis revealed that the five significantly differentially expressed miRNAs were linked to several disease terms. There was an association between the disease terms COPD, asthma, and inflammation and the two downregulated miRNAs (miR-146a-5p and miR-19b-3p), as well as miR-18a-5p, which was upregulated in grain dust exposed groups. In addition, the miR-124-3p, that also was upregulated in the exposed workers, showed association with asthma and inflammation. These findings are particularly interesting, as COPD, asthma, and inflammation are relevant outcomes for grain dust exposed workers^3,25,27^.

All the five differentially expressed miRNAs were associated with the disease terms lung cancer and Non-small cell lung cancer (NSCLC). At present, there are no data that supports an effect of grain dust exposure on lung cancer risk. The occupational exposure to endotoxin in agriculture and cotton industry has on the contrary been reported to exert a protective effect against pulmonary malignancy^28^. However, the mechanisms behind lung cancer development may be shared by other chronic lung diseases of relevance to grain dust, including COPD, asthma, and pulmonary fibrosis^21–23^. Furthermore, disease terms related to circulation disorders such as coronary artery disease, intermediate coronary syndrome, and lacunar stroke were associated with four of the five differently expressed miRNAs. In addition, an association between pulmonary hypertension and hsa-miR-146a-5p was observed. Dust exposure may increase the risk of cardiovascular disease by inducing oxidative stress and activation of inflammatory pathways^29^. Although we did not find any support of increased risk of cardiovascular disease due to grain dust induced platelet activation in a previous study^27^, it is possible that exposure may induce effects at the level of miRNA regulation.

There were three upregulated miRNAs (miR-574-3p, miR-124-3p and miR-18a-5p) among the five identified miRNAs (Fig. 2). Target genes for one of the best predictor miRNA of grain dust exposure, hsa-miR-574-3p, were enriched in KEGG pathways such as MAPK signalling, T-cell receptor signaling, and endocytosis and chemokine signaling. All of this signaling pathways are important pathways in inflammatory and immunological responses, which indeed is relevant for the response to occupational grain dust exposure. The association of miR-574-3p with lung cancer (as shown in Table 5), has been observed in several studies where an upregulation resulted from TLR9 signaling^30^. The involvement of miR-574-3p in migration and invasion regulation in vitro, and metastasis^31^, suggests that miR-574-3p may have a role in epithelial-to-mesenchymal transition (EMT). EMT is a universal mechanism in growth and development, and is also frequently involved in the development of cancer^32^. It is, however, also important in the development of other pulmonary and vascular disorders, the latter in the form of endothelial-to-mesenchymal transistion (EndMT)^33^. Although not directly demonstrated, miRNA mediated alterations in genes involved in EMT may be relevant markers of possible adverse health effects in grain dust workers.

The other miRNA grain dust predictor, miR-124-3p, is reported to be associated with asthma and inflammation, as well as arteriosclerosis and stroke. In our miRNA-disease association analysis, coronary artery disease was one of the disease terms associated with has-miR-124-3p. In human pulmonary artery smooth muscle cells in vitro, hsa-miR-124-3p was shown to target GRB2^34^. The ERK2/GRB2/Shc pathway is critical in vascular smooth muscle cell proliferation, and this indicates that differential expression of hsa-miR-124-3p might be involved in the pathogenesis of pulmonary artery hypertension in patients with COPD. Moreover, the expression level of hsa-miR-124-3p is significantly reduced in pulmonary artery smooth muscle cells from COPD patients with pulmonary artery hypertension. Therefore, hsa-miR-124-3p can be a potential molecular marker for grain dust exposure that is related with adverse effects.

The third grain dust exposure predictor, miR-18a-5p, was associated with COPD, asthma and inflammation disease terms, that are relevant endpoints regarding occupational grain dust exposure. Further, the association of miR-18a-5p with coronary heart disease and arteriosclerosis may also be relevant for grain workers. The miR-18a-5p target genes enriched in chemokine signaling pathway may support mechanisms involved in development of coronary heart disease and arteriosclerosis, since chemokines are considered as markers of atherosclerosis and other cardiovascular diseases. It has been reported that overexpression of miR-18a-5p is associated with (EndMT) and cardiac fibrosis^35^. Furthermore, hsa-miR-18a-5p is involved in NFkappa B signaling pathway, linking this miRNA to inflammation in rheumatoid arthritis^36^, and can directly target interferon regulatory factor 2 (IRF2) in NSCLC cells^37^. The enhancement of IL-6 mediated production of the acute-phase proteins fibrinogen and haptoglobin by hsa-miR-18a-5p activation of the STAT3 inhibitor PIAS3 also support a role in inflammation. It has been shown that miR-18a enhanced the transcriptional activity of STAT3^38^.

There was two downregulated miRNAs (hsa-miR-19b-3p and has-miR-146a-5) among the five identified miRNAs (Fig. 2). The downregulated hsa-miR-19b-3p is associated with similar diseases in MNDR as the upregulated miRNAs. KEGG pathways such as “endocytosis” and “adherens junction” were enriched, and could be regarded as relevant for possible adverse responses to grain dust exposure in the lung epithelium.

MiR-146a-5p has the ability to negatively regulate several pro-inflammatory factors that promote progression of diseases such as COPD and atherosclerosis, including toll-like receptor (TLR4), IL-1 receptor-associated kinase 1 (IRAK1) and TNF-receptor associated protein factor 6 (TRAF6)^39^. MiR-146a-5p is involved in epithelial-fibroblast communication in the lungs, and the pro-inflammatory phenotype of COPD fibroblasts that have been shown to result from dysregulation of epithelial-fibroblast interaction, partly due to the reduced ability of COPD-derived fibroblasts to upregulate miR-146a-5p to counter-regulate pro-inflammatory activity^40^. Furthermore, downregulation of mir-146-a/TLR4 signaling protects against sever burn-induces remote acute lung injury via anit-inflammation^41^. Down-regulation of miR-146a-5p may thus reflect a pro-inflammatory process in grain dust exposed workers.

Fungal induction of IL-6 and TNFα through Dectin-1-Syk-NF-κB and p38MAPK pathways has been associated with increased miR-146, whereas an overexpression of miR-146 inhibited the NF-κB promoter-binding activity, suppressing the IL-6 and TNFα production^42^. MiR-146a has therefore been suggested to act as a potent negative feedback regulator in inflammatory response following Dectin-1 stimulation^42^. It is furthermore shown that hsa-miR-146a-5p can be induced by other microbial components, such as LPS^16^. In the present study, the observed downregulation of hsa-miR-146a-5p in exposed workers versus controls, and the fact that the workers were exposed to high levels of fungal spores and endotoxin, as well as having increased serum IL-6 concentration, supports such a regulatory function of hsa-miR-146a-5p related to microbial exposure and inflammation.

Epigenetic studies in mice models have suggested a role for increased miR-146a in allergic rhinitis. The pulmonary miRNA expression profiles in mice were shown to be altered by sub chronic *Aspergillus fumigatus* exposure^43^. These were other miRNA than the significantly altered circulating serum miRNA in the present study*.* Although miRNAs are well conserved between species and generally show similar target interactions, results on circulating miRNA in mice may not always be relevant for humans.

There is indeed a lack of literature regarding (occupational) bioaerosol exposure, expression of circulating miRNAs, and their role as biomarkers and early predictors of disease. In the present study we could not find any significant relationship between the measured bioaerosol exposure and the miRNAs that were significantly differently expressed in exposed compared to unexposed workers. This may indicate either (1) that very low exposure levels are sufficient for changing the miRNA expression levels; (2) that the exposure measurements are not precise enough to represent the aerosol fraction containing or the specificity of the agent that causes the effect; or (3) that other unknown parameters in the exposed situation, not covered by this study, are of importance for miRNA expression. Investigation of epigenetic effects such as miRNA expression profiling in groups of individuals with differentially occupational exposure can potentially increase the knowledge of exposure–response-mechanisms.

The present study shows that 5 miRNAs differently expressed between exposed workers and the controls could represent important biomarkers of grain dust exposure and may have a potential as early disease markers in exposed workers. As no association between the miRNAs and the personal bioaerosol exposure measurements were found, the specific causative agent(s) of the miRNA changes was not elucidated. A positive correlation between miR-124-3p, miR-18a-5p, and miR-574-3p and IL-6 protein level was shown, while miR-19b-3p was inversely correlated with CC-16 and sCD40L protein levels. Each miRNA have the potential to regulate the expression of multiple mRNA targets, and the five miRNAs identified as differentially regulated in the present study could potentially interfere with multiple signaling pathways. This generates a complicated picture, and additional experimental validation of the use of miRNA as biomarkers should be performed. It can neither be excluded that other circulating miRNA that were not included in the present study also may play important roles in these contexts.

## Methods

### Ethic statement

The Regional Ethical Committee of South-East Norway and the Norwegian Data Inspectorate approved the study. All participants gave their written informed consent upon participation in the study. All but one of the workers that received the written information agreed to participate. The one that refused blood sampling participated in the exposure measurements only. All experiments were performed in accordance with relevant guidelines and regulations.

### Study population

**Figure 4:**
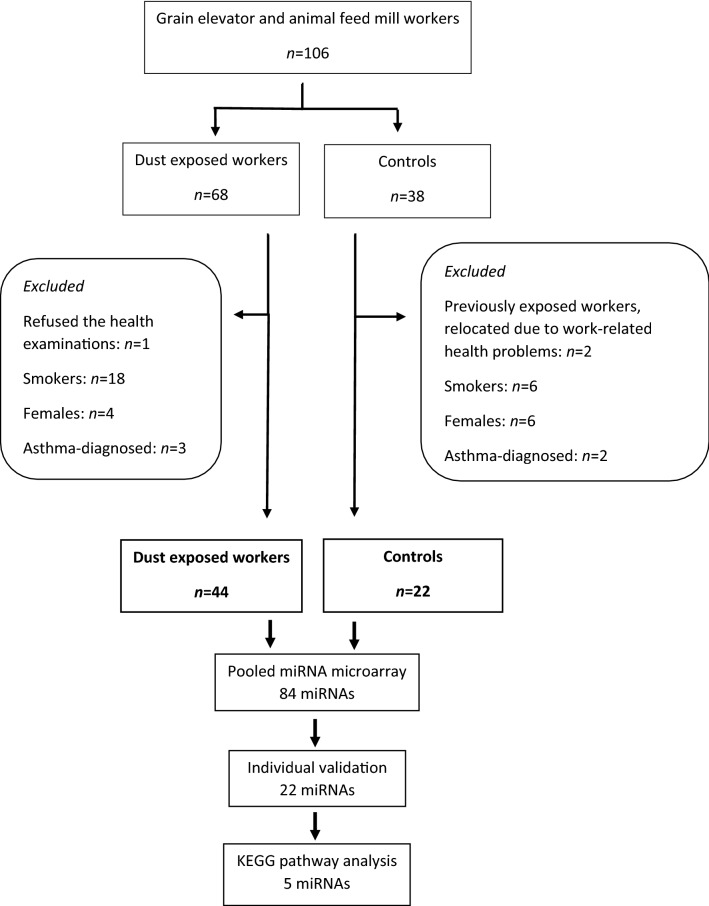
Flow chart of selection of study population and miRNA analyses.

Details of recruitment and description of the study population, participating companies and work tasks carried out by the workers have been published previously^3,44^. Twenty companies of grain elevators and compound feed mills in the Norwegian grain industry geographically distributed throughout 20 municipalities in nine counties in central and south-eastern Norway were included in this study. The study population consisted of all employees working on relevant shifts; 68 grain dust exposed workers and 36 assumedly unexposed administrative workers as controls. The miRNA expression level may be greatly influenced by smoking habits, gender and asthma and may complicate the assessment of the effect of dust exposure on miRNA expression level^26,45^. Therefore, smokers, females and asthma-diagnosed workers were excluded from the miRNA expression analysis. The selected study population consisted of 66 exposed workers and 22 controls (Fig. 4). The exposed workers included 20 workers employed in the grain elevator department, 13 workers in the compound feed mill department, 7 working both in grain elevator and compound feed mill departments, and four transport workers. Characteristics of the selected study population are presented in Table 1.

### Bioaerosol exposure measurements and analyses

Full shift (6–8 h) personal inhalable samples (n = 66) were collected with PAS-6 samplers^46^ and portable pumps (PS101; National Institute of Occupational Health, Oslo, Norway) using a flow rate of 2 L min^−1^. Samples were analyzed for grain dust, endotoxins, bacteria, fungal spores and β-1,3-glucans as previously described^10^. In brief, dust was weighed, bacteria were stained with acridine orange and counted by epifluorescence microscopy, fungal spores were counted by scanning electron microscopy, endotoxins were analyzed by Limulus amoebocyte lysate assay, and β-1,3-glucans were analyzed by enzyme-immuno-assay. The job groups and exposure variability have been described previously^10,44^.

### Blood sampling

Blood samples were collected after work between 1 and 3 p.m. in 2008 and 2009. The blood was collected into vacutainers without additives (BD Vacutainer, Franklin Lakes, NJ, USA), and left to coagulate for 30–90 min at room temperature before isolation of serum. Samples were immediately frozen in aliquots to − 20 °C, replaced to − 80 °C upon arrival at the laboratory, where they were kept until analysis. Serum proteins were analyzed in 2009, whereas the RNA extraction and miRNA analyses were carried out in 2017–2018.

### Analysis of serum proteins

The pneumoproteins and other inflammatory markers in blood were analyzed by enzyme-linked immunosorbent assay (ELISA), as previously described^27^.

### RNA isolation and reverse transcription

Total RNA from blood serum samples were isolated using Qiagen miRNeasy Serum/Plasma kit (Qiagen N.V., Hilden, Germany) according to the manufacturer’s procedure. An initial serum volume of 200 μL were used for all samples. Synthetic *C. elegans* miRNA (cel-miR-39) was used as spike-in control. After RNA extraction and clean-up, the RNA was eluted with RNAse free water and stored at − 80 °C until analysis.

The cDNA synthesis was performed as previously described^47^, using the miScript II RT Kit (Qiagen) according to the manufacturer’s protocol. All cDNA samples were stored at − 20 °C prior to miRNA expression analysis.

### miRNA qPCR-array of pooled serum samples

In an initial miRNA qPCR-array expression profile screening, the following eight group-based cDNA pools were prepared from each individual cDNA sample: (1) exposed workers (*n* = 44), (2) controls (*n* = 22), (3) farm childhood yes (*n* = 16), (4) farm childhood no (*n* = 28), (5) low endotoxin exposure (*n* = 23), (6) high endotoxin exposure (*n* = 21), (7) low fungal spore exposure (*n* = 38), high fungal spore exposure (*n* = 8). Pooled cDNA was pre-amplified using miScript PreAMP PCR Kit and miScript PreAMP Primer Mix (both Qiagen).

A human Serum & Plasma miScript miRNA qPCR Array (Qiagen) containing 84 target miRNA assays was analyzed using miScript SYBR Green PCR Kit (Qiagen) on a QuantStudio 5 Real-Time PCR System (Thermo Fisher Scientific, Waltham, MA, USA).

### Individual validation of serum miRNA by qPCR

Twenty-two miRNA with over ± twofold differential expression between at least two pools in the initial expression profile screening were selected for individual validation of expression levels by qPCR in the 66 un-pooled single samples. These miRNAs were has-miR-100-5p, hsa-miR-103a-3p, hsa-miR-106b-5p, hsa-miR-122-5p, hsa-miR-124-3p, hsa-miR-125b-5p, hsa-miR-145-5p, hsa-miR-146a-5p, hsa-miR-148a-3p, hsa-miR-150-5p, hsa-miR-18a-5p, hsa-miR-191-5p, hsa-miR-19a-3p, hsa-miR-19b-3p, hsa-miR-205-5p, hsa-miR-22-3p, hsa-miR-222-3p, hsa-miR-223-3p, hsa-miR-23a-3p, hsa-miR-24-3p, hsa-miR-423-5p and hsa-miR-574-3p. Four stably expressed miRNAs (hsa-miR-17-5p, hsa-miR-193a-5p, hsa-miR-30d-5p and hsa-miR-16-5p) were selected as reference miRNAs based on results from NormFinder^48^ and GeNorm^49^ evaluation. Each cDNA sample was individually pre-amplified using pooled primers for the selected miRNAs. The real-time qPCR analysis was carried out as previously described^47^ using miScript SYBR Green PCR Kit (Qiagen) on a CFX384 Touch Real-Time PCR Detection System (Bio-Rad, Norway). Serial dilutions of cDNA were prepared to determine the optimal dilution. The cycling program included an initial enzyme activation step at 95 °C for 15 min, and then 40 cycles of denaturation, annealing and extension steps at 94 °C for 15 s, 55 °C for 30 s and 70 °C for 30 s, respectively. Melting curve (Tm) analysis was included in each run. Non-template controls (NTC) were included in each run.

### miRNA data analysis

The quantification cycle (Cq) values were recorded with the Quant Studio 5.0 software (Thermo Fisher Scientific, Norway) or CFX Manager Software (Bio-Rad, Hercules, CA, USA). The qPCR data analysis was performed as previously described^47^ by the comparative Cq-method^50,51^. In brief, prior to normalization, the raw data Cq values were pre‐processed and outliers were excluded from further analysis. In addition, target miRNAs with Cq values > 30 were considered beyond the limit of detection and excluded from further analysis. The PCR efficiency of the miRNAs were estimated using LinRegPCR algorithm^52^ and the raw Cq-values were PCR-efficiency corrected. Then, the PCR-efficiency corrected Cq values were normalized using the geometric average of stably expressed reference miRNAs, [this is given by ΔCq; where ∆Cq (sample) = Cq (target miRNA) − Cq (geometric average of reference miRNAs)], and the ΔCq values were transformed to linear scale [normalized relative quantities (NRQ) = 2^−ΔCq^]. The stability of the reference miRNAs was evaluated by the NormFinder and GeNorm algorithm^49^, and the most stably expressed reference miRNAs were used for normalization. The fold change (FC) between exposed and control samples were then calculated by dividing the average NRQ values of the exposed group samples by the average NRQ value of the control group samples. The miRNA expression level similarity in all individual samples were studied by hierarchical clustering analysis (average linkage and Euclidean distance similarity measurement) using the MeV software version 4.9^53^, and visualized in a dendrogram.

### Statistical analysis

The concentrations of bioaerosols and serum proteins were log_10_-transformed to achieve normal distribution and homoscedasticity. The concentration of bioaerosols are presented as arithmetic mean (AM), geometric mean (GM) and geometric standard deviation (GSD). The serum protein concentrations are presented as GM (GSD). Confounding effects of age, % body fat, farm childhood, living at a farm, and having a cold on the serum protein concentrations were tested by stepwise backward procedure and adjusted for at a *p* value of < 0.1 and when the regression coefficient changed by > 20%. Socio-economic status such as education were not registered or included as a potential confounder. General linear models (GLM) were built to calculate estimates of adjusted serum protein concentrations. Differences in the GM between exposed workers and controls were tested by independent sample t-test, and a *p* value ≤ 0.05 was regarded statistically significant. Pearson’s correlation analyses were used to study the correlations between log-transformed unadjusted values of serum proteins and miRNAs, and between bioaerosol exposure and miRNAs. A *p* value ≤ 0.05 was regarded statistically significant. The association between miRNA expression and the bioaerosol exposure were explored with GLM using both continuous exposure variables and categorical exposure variables consisting of high and low exposure categories. The discriminatory values of miRNAs differentially expressed in exposed workers and controls were assessed by ROC curves with corresponding AUC statistics. The IBM software package SPSS version 25.0 0 (IBM Corp, Armonk, NY, USA) was used for the statistical analyses.

### miRNA target prediction, pathway analysis and disease-association analysis

The target genes of the identified significantly differentially expressed miRNAs were predicted using miRWalk database v2.0^54,55^ which integrated several databases, in order to identify which signaling pathways that might be targeted by these miRNAs. The identified putative target genes were used to predict effects of dust exposure on Kyoto Encyclopedia of Genes and Genomes (KEGG)^56,57^ signaling pathways. Five statistically differentially expressed miRNAs were used in the predictions. The KEGG enrichments analysis is based on hypergeometric statistical tests, including Benjamini and Hochberg (FDR < 0.05) multiple test adjustment. MiRNA-disease associations were performed by extracting disease terms linked to the five identified miRNAs from the MNDR v2.0 (Mammalian ncRNA-disease repository) database, which is a comprehensive tool for efficient extraction of the relationships between diverse ncRNAs and diseases^58,59^. Then miRNA-disease associations with lung-disease related terms were selected, and duplicate associations with conflicting evidence were subsequently removed. The study was approved by the Regional Ethical Committee of South-East Norway, and received support from the Confederation of Norwegian Enterprise (S-2585).
